# Paying for Worms

**DOI:** 10.1371/journal.pntd.0005092

**Published:** 2016-12-29

**Authors:** Peter J. Hotez, Ashish Damania, Mohsen Naghavi

**Affiliations:** 1 Sabin Vaccine Institute and Texas Children’s Hospital Center for Vaccine Development, National School of Tropical Medicine at Baylor College of Medicine, Houston, Texas, United States of America; 2 Department of Biology, Baylor University, Waco, Texas, United States of America; 3 Center for Health and Biosciences, James A Baker III Institute for Public Policy, Rice University, Houston, Texas, United States of America; 4 Institute for Health Metrics and Evaluation, University of Washington, Seattle, Washington, United States of America; New York Blood Center, UNITED STATES

## Abstract

Although approximately one-half of the global disease burden due to the major helminthic infections occurs among the poor living in rich economies, almost all of the public support for helminth control and research and development comes out of the United States and Europe.

The concept known as “blue marble health” arose during the final years of the Millennium Development Goals and in the wake of global health gains in developing countries resulting from massive overseas development assistance [1–3]. Financial support for these programs came from the group of seven (G7) countries (e.g., the Global Fund to Fight AIDS, Tuberculosis and Malaria), in addition to specific funds from the US and the United Kingdom [1–3].

Through such programs and resulting reductions in the deaths from or prevalence of AIDS, tuberculosis, malaria, and neglected tropical diseases (NTDs), a new global health paradigm emerged. A central tenet of blue marble health is that today most of the world’s NTDs, including one-half of the world’s helminth infections, are paradoxically found in the wealthy group of 20 (G20) nations, as well as in Nigeria [1–5]. Thus, with some exceptions, most of the global NTD burden is found among impoverished populations who live in the largest economies. Additional studies find that AIDS, tuberculosis, and malaria also largely occur in the G20 and Nigeria [6], as do noncommunicable diseases (NCDs) [7]. The overwhelming numbers of cases of NTDs and other neglected diseases in G20 countries and Nigeria occur among the poor living in those countries.

An in-depth analysis of the Global Burden of Disease Study 2013 for helminth infections reveals (for the year 2013) that nine worm diseases—food-borne trematodiases, schistosomiasis, hookworm disease, ascariasis, trichuriasis, cysticercosis, echinococcosis, lymphatic filariasis, and onchocerciasis—caused approximately 14.5 million disability-adjusted life years (DALYs) globally [8], including 7.5 million DALYs in G20 countries and Nigeria [9]. As shown in Fig 1, the nations of China, India, and Nigeria accounted for the highest helminth disease burden, followed by Indonesia, South Africa, Brazil, and Mexico. A key reason for the unexpectedly high helminth disease burden in China is due in part to disease burdens resulting from two food-borne trematodiases—clonorchiasis and paragonimiasis—in addition to widespread hookworm and other soil-transmitted helminthiases [10]. In India, hookworm and other soil-transmitted helminthiases together with lymphatic filariasis account for high disease burdens there [11], while in Nigeria schistosomiasis, hookworm and other soil-transmitted helminth infections, lymphatic filariasis, and onchocerciasis represent the major helminth infections [12].

**Fig 1:**
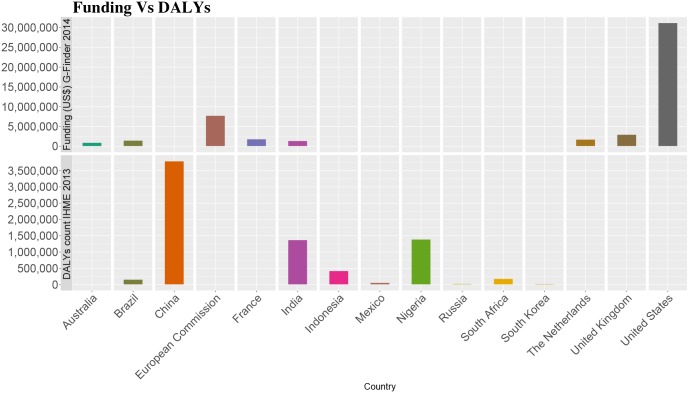
Comparison of (top) public sector—government funding for research and development on helminth infections in G20 countries and Nigeria, based on G-FINDER [13], with (bottom) the DALYs resulting from helminth infections in G20 countries and Nigeria [9].

In contrast to the helminth disease burden, almost all of the research and development (R&D) support for helminth infections in 2014 came from the US, the European Commission, and the UK, followed by France, The Netherlands, Brazil, India, and Australia [13]. In all, approximately US$50 million was spent on helminth R&D via the public sector—governments in 2014 [13]. This information is based on the Policy Cures 2015 G-FINDER Report for the year 2014 and includes basic science R&D and R&D directed towards product development [13]. However, we believe that the figure quoted for Australia may represent an underestimate based on first-hand knowledge of Australian support for some of our colleagues. Moreover, the German Federal Ministry of Education and Research has recently established a new fund to support NTD R&D, which could include helminth infections.

In contrast to the US and European countries, overall the G20 nations with the highest helminth disease burden do not contribute significant R&D funds toward innovations for helminthiasises. G-FINDER reveals that of the helminth-endemic G20 middle-income countries, only the nations of India (US$1.4 million) and Brazil (US$1.5 million) modestly support helminth R&D [13]. Therefore, in the G20 nations where helminth infections are endemic, including Brazil, China, India, Indonesia, South Africa, and Mexico, there is an urgency to increase financial support for R&D. Nigeria is also in a position to begin funding R&D for helminth infections. Outside of public sector funding, the Bill & Melinda Gates foundation contributed the majority of the private philanthropic funding towards helminths with US$23.4 million, followed by the Wellcome Trust with US$5.2 million [13].

The global dependence on the US and Europe for helminth R&D extends to funds for supporting implementation of mass drug administration (MDA) for helminth infections. According to the US Agency for International Development (USAID), the US government through its NTD program provides annual support of approximately US$100 million to support delivery of essential medicines for mass treatments of the intestinal helminth infections, schistosomiasis, lymphatic filariasis, and onchocerciasis [14]. Similarly, the UK Coalition against NTDs reports that the British Department for International Development (DFID) “increased its expenditure from £50 million to £245 million over the four years from 2011–2015” [15]. Moreover, the UK has just launched a new £1 billion Ross Fund jointly with the Gates Foundation to support innovations for NTDs and other serious infections affecting developing countries [16]. Beyond the US and the UK, however, it is not clear how much the other G20 nations, including the helminth-endemic G20 countries, or Nigeria contributes towards MDA. There is an important need to better account for these contributions.

A key policy implication of blue marble health is that the G20 nations need to initiate or expand their contributions to both neglected diseases treatments and R&D. At least for the helminth infections, the gaps are obvious, especially for some of the so-called BRICS (Brazil, Russia, India, China, and South Africa) nations and the Latin American G20 countries—Argentina, Brazil, and Mexico—where endemicity is also high. We also recognize that the G20 countries face high health burdens that go beyond helminth infections and other neglected diseases, and in some cases these countries may have important trade-offs to support NCDs or other conditions.

Financial support for helminth R&D and MDA could become an important theme for future G20 summits. Given previous reports that show an inverse association between the helminth infections (“worm indices”) and human development indices [17], financial commitments for these diseases most certainly fall within the enlightened self-interests of the G20 countries and Nigeria. These issues should be on the agenda at future meetings and summits of the G20 and the United Nations (and its agencies).
